# Betaglycan Is Required for the Establishment of Nephron Endowment in the Mouse

**DOI:** 10.1371/journal.pone.0018723

**Published:** 2011-04-18

**Authors:** Kenneth A. Walker, Sunder Sims-Lucas, Georgina Caruana, Luise Cullen-McEwen, Jinhua Li, Mai A. Sarraj, John F. Bertram, Kaye L. Stenvers

**Affiliations:** 1 Department of Anatomy and Developmental Biology, Monash University, Clayton, Victoria, Australia; 2 Prince Henry's Institute of Medical Research, Clayton, Victoria, Australia; 3 Rangos Research Center, Children's Hospital of Pittsburgh of UPMC, Pittsburgh, Pennsylvania, United States of America; Institut National de la Santé et de la Recherche Médicale, France

## Abstract

Betaglycan is an accessory receptor for the transforming growth factor-β (TGFβ) superfamily, many members of which play key roles in kidney development. The purpose of this study was to define the role of this co-receptor on fetal murine kidney development. Stereological examination of embryonic and adult betaglycan heterozygous kidneys revealed augmented nephron number relative to littermate controls. Fetal heterozygous kidneys exhibited accelerated ureteric branching, which correlated with augmented nephron development at embryonic day (e) 15.5. In contrast, betaglycan null kidneys exhibited renal hypoplasia from e13.5 and reduced nephron number at e15.5. Quantitative real-time PCR analysis of e11.5–e14.5 kidneys demonstrated that heterozygous kidneys exhibited a transient decrease in *Bmp4* expression at e11.5 and a subsequent cascade of changes in the gene regulatory network that governs metanephric development, including significant increases in *Pax2*, *Eya1, Gdnf, Ret, Wnt4,* and *Wt1* expression. Conversely, gene expression in null kidneys was normal until e13.5, when significant reductions were detected in the expression of *Bmp4* as well as other key metanephric regulatory genes. *Tgfb1* and *Tgfb2* mRNA expression was down-regulated in both nulls and heterozygotes at e13.5 and e14.5. The opposing morphological and molecular phenotypes in betaglycan heterozygote and null mutants demonstrate that the levels of betaglycan must be tightly regulated for optimal kidney development.

## Introduction

The development of the metanephros and the subsequent establishment of nephron endowment begins when an outgrowth from the Wolffian duct known as the ureteric bud, extends towards and invades the metanephric mesenchyme [1]. Reciprocal interactions between the epithelial ureteric bud and the surrounding metanephric mesenchyme result in branching and extension of the ureteric tree, which generates the framework of the collecting duct system of the adult kidney. At the tips of the ureteric tree branches, mesenchymal condensation and mesenchymal-to-epithelial transition occur giving rise to nephrons [2].

The many processes involved in metanephric development are tightly regulated by complex molecular regulatory networks [3]. Members of the TGFβ superfamily of signalling molecules have previously been shown both *in vitro* [4], [5], [6] and *in vivo* to regulate key aspects of metanephric development [7], [8], [9], [10], [11], [12]. At embryonic day (e) 11.5, transcripts for the three TGFβ isoforms (*Tgfb1*–*3*), the type I (*Tgfbr1*) and II (*Tgfbr2*) TGFβ signalling receptors as well as transcripts for many other members of the TGFβ superfamily are present in the mouse kidney [11]. Of the TGFβ isoform null mouse lines, only *Tgfb2* mutant mice exhibit defects in kidney development [6], [12], [13], [14]. These include dysplastic ureteric tree formation, impaired glomerulogenesis and renal agenesis [6], [12]. In contrast, *Tgfb2* heterozygous mice exhibit accelerated metanephric development, which results in augmented nephron endowment at postnatal day (PN) 30 [6]. These data indicate that TGFβ2 plays complex, non-redundant roles in the developing metanephros, and further understanding of the factors that control TGFβ2 action in the fetal kidney is required.

Recent evidence suggests that the efficacy of TGFβ isoforms in tissue development depends on the presence or absence of specific TGFβ superfamily co-receptors such as the type III TGFβ receptor (TGFBR3), commonly referred to as betaglycan [15], [16]. Betaglycan presents ligand to the type II TGFβ receptor, an action of particular importance for TGFβ2, which does not bind to the type II TGFβ receptor with high affinity alone [15], [17], [18]. Additional studies have highlighted a more complex role for betaglycan, which is now known also to directly bind inhibins and certain BMPs through its core, while indirectly influencing BMP and activin function through the regulation of inhibins [19], [20].

To define the role of this co-receptor in metanephric development, we studied betaglycan heterozygous (betaglycan^+/−^) and null (betaglycan^−/−^) mouse kidneys at the morphological and molecular levels. We show that betaglycan mutant kidney phenotypes are similar to the previously characterized *Tgfb2* mutant phenotypes. Furthermore, we demonstrate that the opposing phenotypes in the betaglycan^−/−^ and betaglycan^+/−^ kidneys arise from distinct alterations in the positive and negative gene regulatory networks which govern fetal kidney formation. Collectively, our data indicate that the TGFβ2/betaglycan signalling pathway has dosage-dependent actions within the fetal kidney, and that this pathway must be tightly controlled for normal branching morphogenesis and nephrogenesis to occur.

## Results

### Betaglycan is expressed in the developing mouse kidney

**Figure 1:**
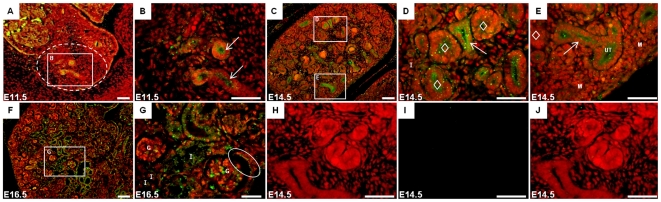
Localisation of betaglycan in the developing mouse kidney. Betaglycan immunostaining (*green fluorescence*) in mouse kidney at e11.5 (A–B); e14.5 (C–E) and e16.5 (F–G). Negative control, e14.5 betaglycan null metanephros (TO-PRO: H, Betaglycan: I, Merge: J). Arrows, ureteric epithelium; M, metanephric mesenchyme; I, interstitial mesenchyme; diamonds, pre-glomerular structures; G, glomeruli; Oval, Bowman's capsule; Red fluorescence, TO-PRO nuclear staining. Scale bars  = 100 µm.

At e11.5, betaglycan was localised to the ureteric epithelium, with no specific immunostaining detected in the metanephric mesenchyme (Figure 1A–B). By e14.5, betaglycan was detected in developing pre-glomerular structures while remaining strongly expressed in ureteric epithelium (Figure 1C–E). At this developmental stage, expression was also observed in the interstitium (Figure 1D) but was absent from the mesenchyme in the nephrogenic zone (Figure 1E). At e16.5, betaglycan immunolocalization was similar to that seen at e14.5 (Figure 1F–G). Betaglycan expression was not detected in the negative control, e14.5 betaglycan null kidney (Figure 1H–J).

### Contrasting developmental phenotypes in betaglycan mutant metanephroi

Stereological analysis of e13.5 metanephroi identified a significant 30–40% decrease in metanephric volume in betaglycan^−/−^ embryos compared to wildtype and betaglycan^+/−^ embryos (Table 1). Likewise, absolute ureteric epithelial volume was significantly decreased in betaglycan^−/−^ mice (Table 1). However, the relative volume of the ureteric epithelium was comparable in all three groups. At e15.5, metanephric volume in betaglycan^+/−^ was significantly greater (30%) than in wildtype embryos, which in turn were 49% larger than the kidneys of betaglycan^−/−^ embryos (Table 1). However, no differences in total kidney weights or volumes were observed between wildtype and betaglycan^+/−^ at PN30 (Table 2).

**Table 1:** Stereological data for betaglycan wildtype, heterozygous and knockout mouse kidneys at e13.5 and e15.5.

		+/+	+/−	−/−	*p*
**e13.5**	**V_kid_** (mm^3^)	0.109±0.018^a^	0.103±0.021^a^	0.077±0.011^b^	<0.001
	**V_ue_** (mm^3^)	0.015±0.003^a^	0.015±0.003^a^	0.010±0.001^b^	0.0013
	**% V_ue of_ V_kid_**	13.50±1.67^a^	13.56±0.39^a^	12.58±0.26^a^	0.3
**e15.5**	**V_kid_ (mm^3^)**	0.261±0.034^a^	0.340±0.055^b^	0.187±0.005^c^	<0.001
	**No. of PNA^+ve^ Structures**	68±11^a^	90±12^b^	47±8 ^c^	<0.001

Values are mean ± S.D. e13.5: (n)  =  +/+ (5), +/− (9), −/− (6); e15.5: (n)  = 7 mice/genotype. Data were analysed via one-way ANOVA followed by a Tukey's post-hoc analysis. Those groups not sharing a common letter are significantly different (*p*<0.01).

**Table 2:** Stereological analysis of female wildtype (+/+) and betaglycan heterozygous (+/−) mouse kidneys at postnatal day 30.

	+/+	+/−
**Body weight** (g)	16.52±1.66	16.46±1.69
**Kidney weight** (g)	0.120±0.024	0.123±0.020
**Kidney volume** (mm^3^)	94.62±8.87	101.37±18.35
**Total glomerular number**	11395±921	13960±693**
**Mean glomerular volume** (×10^−4^ mm^3^)	1.678±0.151	1.316±0.226*
**Total glomerular volume** (mm^3^)	1.910±0.375	1.844±0.370
**Mean renal corpuscle volume** (×10^−4^ mm^3^)	2.273±0.334	1.795±0.320*
**Total renal corpuscle volume** (mm^3^)	2.586±0.341	2.515±0.523

Values are mean ± S.D. (n)  = 7 mice/genotype,

*p<0.05,

**p<0.0001.

### Betaglycan^+/−^ mice have augmented nephron endowment at e15.5 and PN30

Analysis of nephron number at e15.5 revealed that betaglycan^+/−^ kidneys contained approximately 32% more PNA-positive structures than wildtype metanephroi (Table 1). In contrast, betaglycan^−/−^ metanephroi contained 32% fewer PNA-positive structures than wildtype kidneys, and approximately half the number as betaglycan^+/−^ kidneys (Table 1). At PN30, kidneys from betaglycan^+/−^ mice contained 13,960±693 nephrons compared with 11,395±921 nephrons in age-matched wildtype kidneys (Table 2), representing a 23% increase in total nephron number in betaglycan^+/−^ mice (*p*<0.0001). Augmented nephron endowment was accompanied by significant decreases in mean glomerular volumes (p<0.05). However, total glomerular and renal corpuscle volumes were similar in the two groups (Table 1). No renal histopathology was observed in either genotype at PN30 (data not shown).

### The length of nephrogenic period is normal in betaglycan heterozygote mice

**Figure 2:**
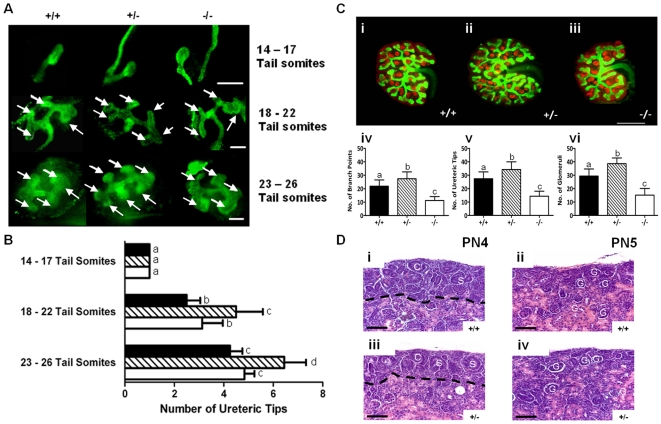
Effect of decreased endogenous betaglycan expression on metanephric development and glomerulogenesis. (A) Representative images of developing metanephroi from wildtype (+/+), betaglycan heterozygous (+/−) and null (−/−) embryos with 14–17 tail somites (e11.0), 18–22 tail somites (e11.5) and 23–26 tail somites (e12.5). Arrows, ureteric tips; scale bars  = 150 µm. (B) Total number of ureteric tips present in +/+ (solid bars), +/− (striped bars) and −/− (clear bars) developing metanephroi, at progressive developmental stages. n≥5 genotype/stage. (C) Wholemount immunofluorescent labelling of the ureteric tree (Calbindin D28K, *green fluorescence*) and developing glomeruli (WT1, *red fluorescence*) in e12.5 betaglycan wildtype (+/+), heterozygous (+/−) and null (−/−) metanephroi following 48 h culture. (iv-vi) Quantification of ureteric branch points, ureteric tips and glomeruli in betaglycan wildtype, heterozygous and null mice (n)  =  +/+ (12), +/− (14), −/− (10). Scale bar  = 250 µm. (D) Representative histological images of PN4 and PN5 kidneys from wildtype (+/+) and betaglycan heterozygous (+/−) mice (i-iv). Comma-shaped (C) and S-shaped (S) bodies in a clearly defined nephrogenic zone (dotted line) (i,iii), or displaying mature glomeruli (G) and no nephrogenic zone indicating cessation of nephrogenesis (ii,iv). Scale bars  = 100 µm. Values are mean ± SD. Analysis via one-way ANOVA followed by Tukey's post-hoc analysis, across all genotypes and ages. Significant differences (*p*<0.05) between groups are represented by different letters.

Analysis of e11.0 – e12.0 gonadal ridges indicated that the emergence of the ureteric bud from the Wolffian duct commenced in all three genotypes at the same embryonic stage (e11.0) (Figure 2A–B). Between e11.5 and e12.0, the ureteric bud from betaglycan^−/−^ embryos exhibited the same number of branches as wildtype ureteric bud. However, betaglycan^+/−^ metanephroi consistently exhibited an augmented number of ureteric tips at this time, indicating extra ureteric branching had occurred (Figure 2A–B). *In vitro* culture of whole metanephroi from e12.5 betaglycan mutant and wildtype embryos indicated that ureteric branch (*p*<0.01) and tip number (*p*<0.01) were both significantly greater in betaglycan^+/−^ mice (27.43±5.08: 34.29±5.68) than in wildtypes (21.83±4.69: 27.42±5.11; Figure 2C). The increased ureteric tree development in betaglycan^+/−^ mice correlated with a significant increase (*p*<0.01) in the number of developing glomeruli (38.64±4.22) compared with wildtype metanephroi (29.42±5.26). In contrast, betaglycan^−/−^ metanephroi exhibited significantly fewer ureteric branches (11.20±2.94; *p*<0.01), tips (14.40±3.72; *p*<0.01) and glomeruli (15.20±4.92; *p*<0.01) than both wildtype and betaglycan^+/−^ metanephroi. Finally, histological analysis of neonatal kidneys revealed that nephrogenesis was complete in wildtype and betaglycan^+/−^ mice by PN5, as indicated by absence of a nephrogenic zone (Figure 2D).

### Metanephric signalling networks are disrupted in betaglycan mutant mice

#### Early Ureteric Branching Morphogenesis

**Figure 3:**
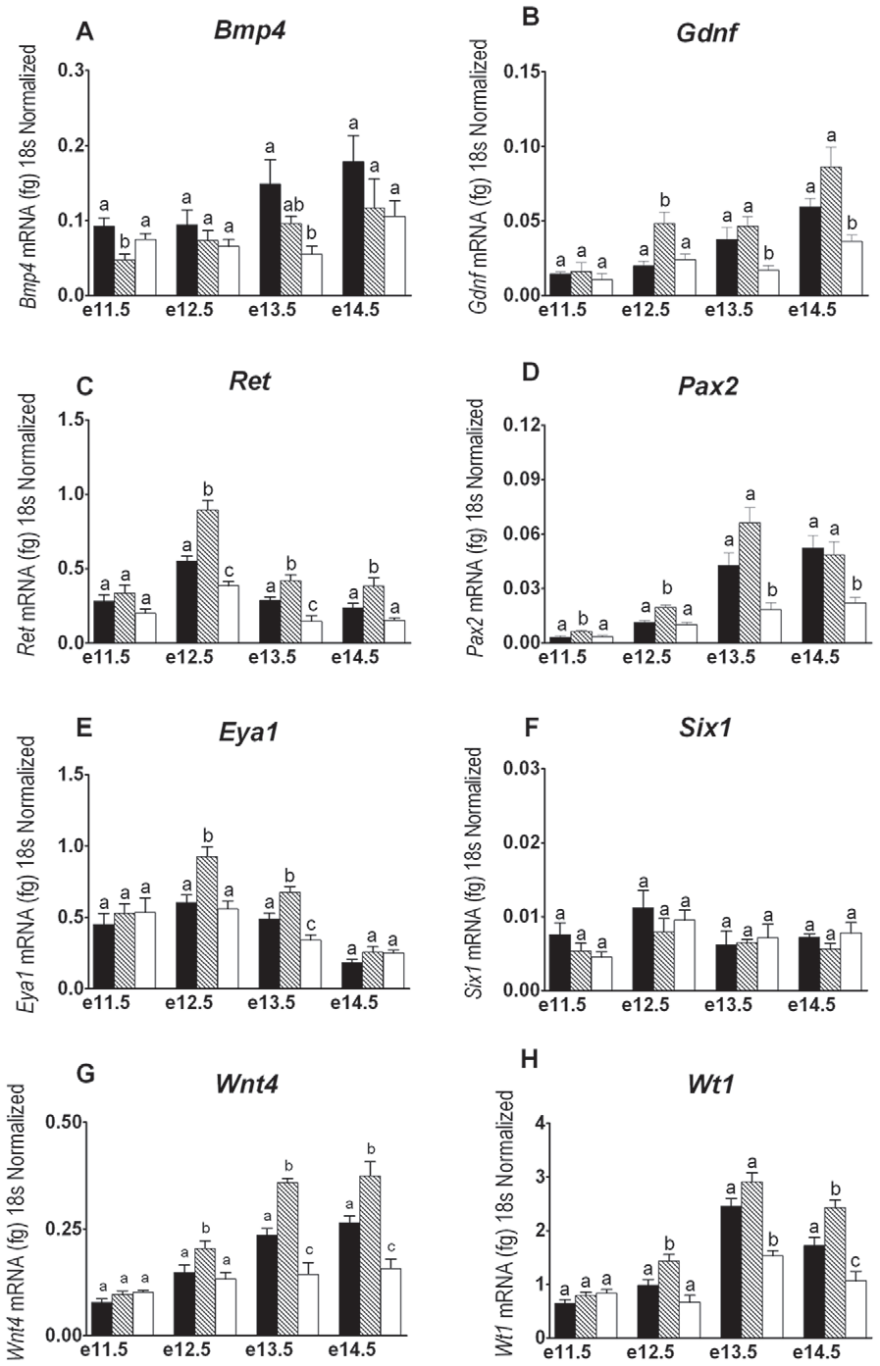
Betaglycan mutant renal mRNA expression. Quantitative real time PCR assessment of genes involved in metanephric development using RNA derived from age-matched wildtype (solid bars), betaglycan heterozygous (striped bars) and betaglycan null (clear bars) metanephroi. Values are mean + SEM. n = 4–6 RNA pools/genotype/age. Analysis via one-way ANOVA followed by Tukey's post-hoc analysis conducted separately for each gene at each time point. Significant differences (*p*<0.05) between genotypes are represented by different letters.

BMP4 and GDNF, both members of the TGFβ superfamily, play important roles in regulating ureteric budding and ureteric branching morphogenesis. Formation of the ureteric bud at approximately e11.0 and subsequent ureteric branching morphogenesis both require secreted GDNF from the metanephric mesenchyme to bind with its cognate receptor RET, as well as inhibition of BMP4 at the ureteric bud site and around the ureteric tips. In accordance with the increased ureteric branching observed in wholemount urogenital ridges, betaglycan^+/−^ mice exhibited significantly decreased *Bmp4* expression at e11.5 (*p*<0.01) while *Gdnf* expression was significantly upregulated at e12.5 in comparison to age-matched wildtype metanephroi (Figure 3A–B). These changes in betaglycan^+/−^ metanephroi were accompanied by consistent increases in *Ret* expression from e12.5 (Figure 3C). In contrast, betaglycan^−/−^ metanephroi displayed wildtype-like mRNA expression profiles for *Bmp4*, *Gdnf* and *Ret* at e11.5 (Figure 3A–C). However, from e12.5-e14.5, significant decreases in the expression of both the inhibitory (*Bmp4*) and stimulatory pathways (*Gdnf/Ret*) controlling renal branching morphogenesis were detected in betaglycan^−/−^ kidneys (Figure 3A–C).

#### Regulation of the *Ret* and GDNF Expression Profile

GDNF signalling through RET is viewed as the critical positive factor in branching morphogenesis [21], [22]. Multiple studies have identified upstream transcriptional regulators of Gdnf such as Pax2, Eya1 and Six1 as integral to the induction of GDNF expression and in turn early metanephric development [23], [24], [25], [26]. The most important of these factors appears to be Pax2, as Pax2 is not only critical for the initiation of Gdnf expression but also in the enhancement of Ret expression in the developing ureteric tree [23]. At e11.5, Pax2 expression, but not Eya1 or Six1 expression, was significantly increased in betaglycan^+/−^ mutants (Figure 3D–F). At e12.5 and e13.5, betaglycan^+/−^ metanephroi displayed significant increases in both Pax2 and Eya1 mRNA compared with age-matched wildtype, with expression for both genes returning to wildtype levels at e14.5 (Figure 3D–E). Betaglycan^−/−^ metanephroi displayed wildtype-like expression profiles of Pax2 and Eya1 until e13.5, after which time significant decreases in expression of these genes was observed in betaglycan null metanephroi compared to age-matched wildtype metanephroi.

#### Nephrogenesis

Wilm's Tumor 1 (WT1) and Wingless-related MMTV integration site 4 (WNT4) are both essential for nephrogenesis [24], [25], [27], [28]. Changes in mRNA expression for Wt1 were observed from e12.5 with betaglycan^+/−^ metanephroi displaying consistently increased Wt1 expression and betaglycan^−/−^ metanephroi consistently decreased Wt1 expression from e13.5, compared with age-matched wildtype metanephroi (Figure 3H). Similar changes were observed in Wnt4 expression levels (Figure 3G). These data suggest that dysregulation of glomerular formation in betaglycan mutant mice is present from the commencement of glomerulogenesis.

#### TGFβ Isoforms

**Figure 4:**
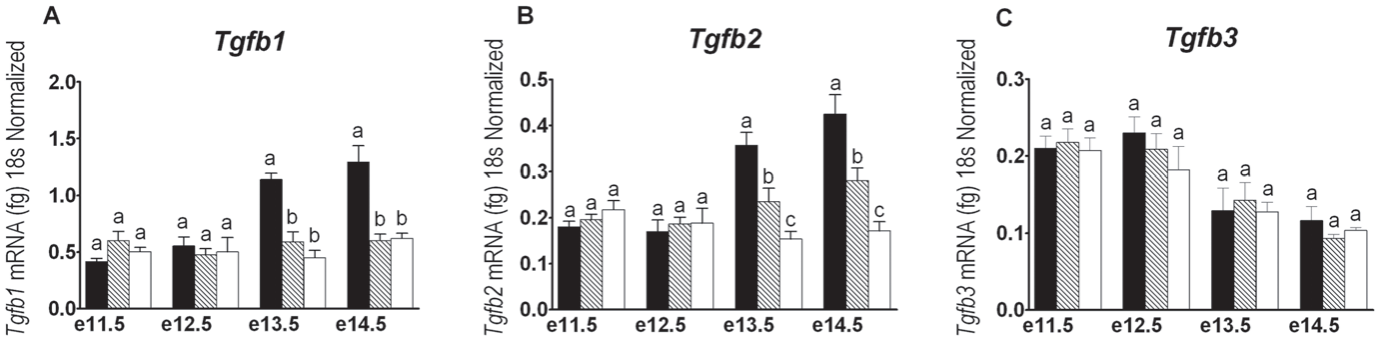
Expression of TGFβ isoforms in kidneys of betaglycan mutant mice. Quantitative real time-PCR assessment of (A) *Tgfb1*; (B) *Tgfb2*; and (C) *Tgfb3* using RNA derived from age-matched wildtype (solid bars), betaglycan heterozygous (striped bars), and betaglycan null (clear bars) metanephroi. Values are mean + SEM. n = 4–6 RNA pools/genotype/age. Analysis via one-way ANOVA followed by Tukey's post-hoc analysis conducted separately for each gene at each time point. Significant differences (*p*<0.05) between genotypes are represented by different letters.

All three TGFβ isoforms were detected in wildtype metanephroi from e11.5, with an upregulation in Tgfb1 and Tgfb2 expression detected at e13.5–e14.5 (Figure 4). This upregulation in Tgfb1 and Tgfb2 mRNA was not observed in betaglycan mutant metanephroi, with both betaglycan^+/−^ and betaglycan^−/−^ mutants displaying decreased expression levels of these genes compared to wildtype littermates (p<0.01; Figure 4A–B). Interestingly, the levels of Tgfb2 and betaglycan mRNA expression appeared to be directly correlated, as Tgfb2 expression at e13.5 and e14.5 was less in betaglycan^+/−^ mutants than in wildtype mice, and less again in betaglycan^−/−^ mutants (Figure 4B). Tgfb3 expression was similar in all three genotypes at all ages examined (Figure 4C).

### SMAD1 and SMAD3 signalling is unchanged in betaglycan mutants

**Figure 5:**
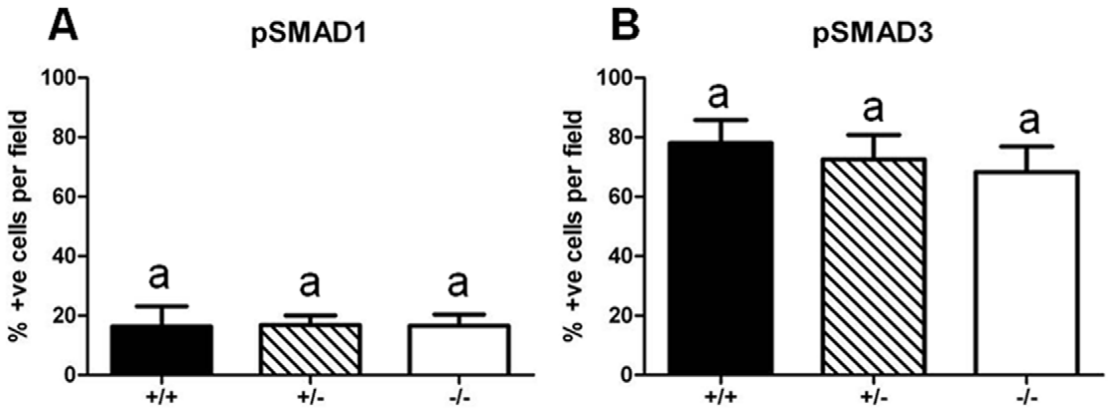
SMAD1 and SMAD3 activation in betaglycan mutant metanephroi. Quantitative analysis of nuclear pSMAD1 and pSMAD3 expression per microscopic field in wildtype (solid bars), betaglycan heterozygous (striped bars) and betaglycan null (clear bars) metanephroi. n = 4 metanephroi per genotype, with a minimum of 5 fields of view per metanephros. Values are mean + SD. Analysis via one-way ANOVA followed by Tukey's post-hoc analysis. No significant differences were found between genotypes for either pSMAD.

Transduction of TGFβ superfamily signals involves the phosphorylation of intracellular receptor associated proteins known as SMADs. SMAD2 and SMAD3 are predominantly associated with activation of TGFβ/activin signalling pathways while BMP/GDF signalling predominantly utilizes SMAD1/5/8 [29], [30]. As betaglycan can bind both TGFβ and BMP ligands, the altered expression of the co-receptor has the potential to disrupt both pathways [20], [31]. To determine whether TGFβ or BMP signal transduction was altered in betaglycan mutants, phospho (p)SMAD1 and pSMAD3 immuno-stained cells were counted in e13.5 metanephroi from betaglycan wildtype and mutant embryos. This analysis illustrated that far fewer cells exhibited pSMAD1 than pSMAD3 in all three genotypes. However, no differences in the percentages of cells containing activated pSMAD1 or pSMAD3 were found between genotypes (Figure 5A–B).

## Discussion

Despite considerable functional redundancy amongst TGFβ superfamily members in the developing kidney [11], morphological and molecular defects were detected in betaglycan^+/−^ and betaglycan^−/−^ kidneys. Tightly controlled betaglycan expression is essential for normal metanephric development as partial or complete ablation of expression results in opposing renal phenotypes. In the betaglycan^+/−^ kidney, changes in gene expression were observed as early as e11.5. Notably, a decrease in *Bmp4* expression at this time was observed in betaglycan^+/−^ kidneys compared with wildtype and betaglycan^−/−^ kidneys. Although BMP4 appears to have multiple roles in kidney development [32], at the time of ureteric bud outgrowth, BMP4 activity in the sleeve of metanephric mesenchyme surrounding the Wolffian duct must be antagonized by an endogenous inhibitor, gremlin 1, for budding to occur [10], [33]. While the timing of ureteric budding appears similar in the three genotypes, BMP4 can also act as an inhibitor of GDNF/RET/WNT-mediated branching morphogenesis [10]. Therefore, the reduced expression of *Bmp4* at e11.5 is in accordance with the observed increase in ureteric branching at this time. Interestingly, the level of renal *Bmp4* mRNA expression in betaglycan^+/−^ mice after this time was not different to that of age-matched wildtype mice. However, a cascade of changes in the gene regulatory network governing metanephric development followed in the betaglycan^+/−^ kidney, including significant increases in expression levels of *Pax2*, *Eya1, Gdnf, Ret, Wnt4,* and *Wt1*. Of these genes, *Pax2* was the first to be upregulated in the betaglycan^+/−^ kidney at e11.5, in keeping with its role in inducing expression of downstream mediators of branching morphogenesis such as *Gdnf* and *Ret* [23], [34].

In contrast to betaglycan^+/−^ kidneys, the rate of early branching morphogenesis in betaglycan^−/−^ metanephroi resembled that of wildtypes. Accordingly, no differences in levels of gene expression were found between null and wildtype kidneys at e11.5. At e12.5, betaglycan^−/−^ kidneys exhibited only small decreases in *Ret* expression compared to wildtypes. However, at e13.5, expression levels of several integral nephrogenic genes were significantly reduced in betaglycan^−/−^ kidneys compared to wildtype, and these reductions correlated with the reduced kidney volume observed in betaglycan^−/−^ embryos at this time. These findings indicate that a complete loss of betaglycan results in uncoupling of the reciprocal inductive signalling pathways that operate between the metanephric mesenchyme and ureteric epithelium to drive the progression of branching morphogenesis and nephrogenesis. In particular, genes that drive the *Gdnf/Ret* regulatory pathway, such as *Pax2,* fail to be upregulated in the betaglycan^−/−^ kidney, which may be a possible mechanism for the development of the hypoplastic renal phenotype at e13.5.

Importantly, the expression of *Six1*, an upstream regulator of *Gdnf*, of similar hierarchal order to *Eya1*, remained similar between all genotypes through out all developmental stages analysed. Previously, direct interactions between *Eya1* and *Six1* have been shown to be essential for the induction and upregulation of *Gdnf* expression in the MM [26], [35]. As *Eya1*, and not *Six1* expression was altered in betaglycan mutant metanephroi, it is possible to suggest that the influence of betaglycan on downstream *Gdnf* expression may be elucidated through perturbations to *Eya1* expression and not that of *Six1*. It is also possible that the conserved *Six1* expression in betaglycan mutants may be responsible for sufficient *Gdnf* expression induction to prevent the manifestation of more severe renal phenotypes such as renal agenesis in betaglycan^−/−^ embryos [35].

Finally, loss or reduction of betaglycan expression resulted in decreased *Tgfb1* and *Tgfb2* expression in the fetal kidney. This may be due to reduced TGFβ sensitivity in the betaglycan mutant kidneys as TGFβs are well-recognized to auto-induce their own expression by both transcriptional and post-transcriptional mechanisms [36], [37]. A loss or reduction in betaglycan on the cell surface may lead to decreased sensitivity to the TGFβs, which could in turn reduce the level of TGFβ auto-induction and compound the effects of the loss of betaglycan. Indeed, in betaglycan^−/−^ kidneys, the collective reduction of several TGFβ superfamily inputs (TGFβ1, TGFβ2, BMP4) coupled with the complete absence of betaglycan, a key determinant of cellular TGFβ sensitivity, may result in sub-threshold levels of TGFβ superfamily signalling, which may explain why betaglycan^−/−^ kidneys exhibit hypoplasia rather than an even greater rate of kidney development than seen in betaglycan^+/−^ mice.

### Is betaglycan essential as a TGFβ co-receptor in the fetal kidney?

As fetal metanephroi express several potential ligands of betaglycan, the specific mechanisms by which diminished betaglycan expression impacts kidney development are not yet clear. However, several pieces of evidence suggest that betaglycan is serving an essential role as a TGFβ2 co-receptor during early kidney development. Firstly, betaglycan expression is coincident with the previously published expression pattern of TGFβ2 in murine metanephroi [4], [11], [38]. Betaglycan was localized in the ureteric epithelium from the earliest stages of ureteric bud formation, and from e14.5 was also detected in metanephric mesenchyme and the epithelium of developing nephrons. TGFβ2 is similarly expressed by the ureteric epithelium, with expression also detected in neighbouring tissues after e14.5 [38]. The expression of betaglycan in the ureteric epithelium at e11.0–e11.5 and the increased branching of the ureteric tree at e11.5–e12.0 suggests that TGFβ2 initially acts directly upon ureteric epithelium to inhibit branching. Subsequent expression of betaglycan in interstitial cell populations from e14.5 suggests that after this age, TGFβ2/betaglycan signalling is involved in the development of both the epithelial and interstitial cell populations of the metanephros.

Secondly, betaglycan and *Tgfb2* mutant renal phenotypes share several similarities. Like betaglycan^+/−^, *Tgfb2* heterozygotes develop normally and possess more nephrons than wildtype mice at PN30, displaying an even greater enhancement of nephron number than betaglycan^+/−^ mice [6], [39]. This suggests that TGFβ2/betaglycan signalling is required to negatively regulate nephrogenesis, possibly through the regulation of ureteric branching morphogenesis. This observation is in agreement with previous work that suggests abrogation of endogenous betaglycan *in vitro* attenuates endogenous autocrine and/or paracrine TGFβ-mediated negative regulation of lung branching morphogenesis [40]. In contrast to the renal phenotype in betaglycan^+/−^ mice, *in vivo* stereological analysis and *in vitro* culture of betaglycan^−/−^ metanephroi revealed a hypoplastic renal phenotype that closely resembled the defective development of *Tgfb2* null explants [6]. These contrasting phenotypes in betaglycan^+/−^ and betaglycan^−/−^ kidneys suggest that the TGFβ2/betaglycan signalling pathway plays dual roles *in vivo*, both limiting the growth of the ureteric tree while supporting the maintenance of the newly-formed nephrons.

The contrasting and complex renal phenotypes in betaglycan^+/−^ and betaglycan^−/−^ mice suggest that TGFβ2/betaglycan signalling is required at more than one stage of kidney development and in more than one cell type, with specific developmental processes in the kidney having higher thresholds for TGFβ2 signalling than others. Specifically, the data suggest that greater TGFβ2 signalling is necessary to limit early branching of the ureteric tree, and that betaglycan is an essential component of the TGFβ2 signalling complex that allows critical thresholds of TGFβ2 signalling to be met during this process. In contrast, TGFβ2-mediated survival of ureteric epithelium during late gestation appears to occur at a lower threshold of TGFβ2 signalling. This conclusion is based on the observation that newly-formed epithelial structures are maintained when the level of either the ligand or the accessory receptor is reduced, as in *Tgfb2* heterozygotes or betaglycan^+/−^ mice, respectively. Conversely, ablation of TGFβ2 signalling in *Tgfb2* null metanephroi results in degeneration of these newly-formed structures, an effect that could be hypothesized to be due to insufficient pro-survival signalling attributed to TGFβ2 [12], [41], [42]. As degeneration of renal tissues was not observed in betaglycan^−/−^ metanephroi, this suggests that the relatively low, but still present TGFβ2 signalling in betaglycan^−/−^ metanephroi is sufficient to satisfy a threshold necessary for cell survival. This further suggests that the processes of ureteric branching morphogenesis and cell/tissue survival have differing TGFβ2 concentration-dependent signalling thresholds.

The betaglycan mutant phenotypes largely resemble their *Tgfb2* mutant counterparts, suggesting that a major role for betaglycan in the fetal murine kidney is as a determinant of TGFβ2 efficacy. Furthermore, the opposing phenotypes in both *Tgfb2* and betaglycan, null and heterozygote kidneys indicate that the TGFβ2/betaglycan signalling pathway has dose-dependent actions and that activity of this pathway must be tightly controlled for normal branching morphogenesis and nephrogenesis to occur. Molecular analysis of betaglycan mutant kidneys indicated that the distinct morphological phenotypes in the betaglycan^+/−^ and betaglycan^−/−^ kidneys were mirrored at the molecular level. In particular, specific temporal changes in the expression of certain TGFβ superfamily members (for example *Bmp4* during the initiation of ureteric branching morphogenesis in betaglycan^+/−^ metanephroi) may underlie the contrasting phenotypes. These findings may be of considerable importance to human health in that renal hypodysplasia is a recognized cause of paediatric renal failure and a risk factor for hypertension in adults [43].

## Materials and Methods

### Animal Models

All animal handling and experimental procedures were approved by the Monash Medical Centre Animal Ethics Committee (Study approval ID B08/26). Wildtype, betaglycan^+/−^ and betaglycan^−/−^ embryos were obtained from timed matings of betaglycan^+/−^ mice, and genotyped as previously described [15], [44]. Embryo development was verified via Theiler staging criteria and the total number of tail somites present from tail to hind limb [45]. Kidneys were dissected from e11.5–e16.5 embryos, processed and fixed as described below. To assess postnatal renal morphology, kidneys were collected from wildtype and betaglycan^+/−^ PN4, PN5, PN6 and PN30 mice, immersion-fixed in 10% neutral buffered formalin, processed and embedded in paraffin wax. Sections (5 µm) were stained with haematoxylin and eosin, periodic acid Schiff's, Masson's trichrome or silver methenamine histological stains as indicated, and assessed in a blind manner.

### Wholemount and section immunolabelling

Metanephroi from wildtype embryos were fixed in 4% paraformaldehyde at 4°C for 30 min, embedded in paraffin wax and sectioned at 5 µm. Immunofluorescence detection was conducted as previously described [44] using primary antibodies against betaglycan (1∶50, R&D Systems, MN, USA), phosphorylated (p)SMAD1 (1∶100, Santa Cruz, CA, USA) or pSMAD3 (1∶100, Abnova, NSW, Australia) in 1% BSA. Betaglycan expression was detected using a donkey anti-goat Alexa Fluor 488-conjugated secondary antibody (1∶400; Molecular Probes). Quantitative assessment of pSMAD1 and pSMAD3 expression was performed by counting positive cells in six non-overlapping high-power (×400 magnification) fields per metanephric section. Results are expressed as the percentage of pSMAD1 or pSMAD3 immunopositive cells per total number of metanephric cells.

For ureteric bud analysis, wholemount Calbindin D_28k_ (Sigma-Aldrich, NSW, Australia) immunofluorescence was performed, as detailed by [46], on gonadal ridges isolated from e11.0 (14–17 tail somites), e11.5 (18–22 tail somites) and e12.0 embryos (23–26 tail somites) [45], [47]. The presence and morphology of the ureteric bud was correlated with the number of developing somites observed. To assess *in vitro* development of the ureteric tree, whole metanephroi from e12.5 embryos were cultured on polycarbonate filters (3 µm pores) in wells containing serum-free Dulbecco's Modified Eagle's Medium/Nutrient F-12 Ham culture medium (350 µl; Sigma-Aldrich) at 37°C and 5% CO_2_, for 48 h. The explants were processed for immunolabeling as above, using antibodies against Calbindin D_28K_ (1∶200; secondary antibody - Alexa Fluor 488 goat anti-mouse, Molecular Probes Inc., OR, USA) and WT1 (1∶100, Santa Cruz; secondary antibody - Alexa Fluor 594 goat anti-rabbit) in combination to detect ureteric epithelium and glomeruli, respectively. The total number of ureteric branch points, branch tips and developing glomeruli were quantified as previously described [6], [48].

### Estimation of nephron number in developing kidneys

To determine the total number of developing nephrons in embryonic kidneys, metanephroi collected from e15.5 mouse embryos were immersion fixed in 4% PFA for 30 min, embedded in paraffin wax and exhaustively sectioned at 5 µm. All sections were blocked in 2% bovine serum albumin (BSA) in PBS with 0.3% Triton-X for 30 mins at room temperature. Sections were then digested with 0.1% neuraminidase (Sigma Aldrich) in 1% CaCl_2_ in PBS, and incubated overnight at 4°C with biotinylated peanut agglutinin (PNA; Sigma-Aldrich) diluted at 20 µg/ml in 2% BSA/PBS and 0.3% Triton-X with 1 mmol/L CaCl_2_/MnCl_2_/MgCl_2_. Following incubation, sections were rinsed twice with PBS. The biotinylated PNA was visualized using the Elite streptavidin/biotin amplification ABC kit (Vector Laboratories Inc., Burlingame, CA, USA). The ABC solution was applied for 1 hour at room temperature, after which slides were washed and the stain developed with diaminobenzidine (DAB; Sigma Aldrich)/0.01% H_2_O_2_/PBS. Sections were counterstained with haematoxylin.

Section pairs were used to estimate PNA-positive developing nephrons using the physical disector/fractionator combination [49]. PNA-positive structures (which included all developing nephrons from early S-shaped bodies to developing glomeruli) were marked in the first (n) section. The nth+5 section was then viewed, with positive structures present in nth and not the nth+5 section counted. Those counted in the nth+5 section and not in the nth section were also counted to double the efficiency of the technique. This process was repeated with the first nominated nth+5 section denoted the nth section and a new nth+5 section selected. Total number of developing nephrons was determined as the total number of PNA-positive structures observed (Cullen-McEwen et al., submitted).

### Estimation of ureteric epithelial volume

Ureteric epithelial volume was determined in e13.5 metanephroi. Paraffin-embedded metanephroi were exhaustively sectioned at 5 µm, after which all sections were dewaxed, rehydrated and blocked in 2% BSA in PBS with 0.3% Triton-X for 30 min at room temperature. Sections were then incubated overnight at 4°C with biotinylated *dolichos biflorus* (DBA; Sigma-Aldrich). DBA-positive structures were visualized using the Elite streptavidin/biotin amplification ABC kit (Vector Laboratories Inc, CA, USA). Unbiased stereology was then used to determine total and relative epithelial volumes, as well as total metanephric volumes [46].

### Estimation of total nephron number at PN30

Total nephron number was determined in female betaglycan heterozygous and wildtype kidneys at PN30. Body weights were recorded and kidneys dissected, cleaned of fat and blood vessels, decapsulated and weighed. Kidneys were immersion-fixed in 10% neutral buffered formalin, and processed for embedding in glycolmethacrylate. Kidney volume, total glomerular number, mean and total glomerular and mean and total renal corpuscle volumes were estimated as described previously [6], [49], [50], with total nephron number estimated using the unbiased physical disector/fractionator combination.

### Quantitative real-time PCR

Total RNA was isolated from pooled e11.5–e14.5 metanephroi and complementary DNA was synthesized as previously described [51]. All findings were observed across four separate, independently collected pools of kidneys for each genotype and age (n = 4–6/pool). Real-time PCR was performed using the Applied Biosystems ABI 7900 HT Fast real-time machine with all reactions performed in triplicate. Yields were converted to femtograms and normalized to the *18S* mRNA level per sample, as previously described [51]. Primers and their optimized annealing temperatures are listed in Table 3. Cycling conditions comprised 10 min denaturation at 95°C, then 55 cycles of denaturing (95°C for 15 sec), annealing (5 sec) and elongation (72°C for 8 sec and 79°C for 8 sec). PCR products were detected at 16–30 cycles. All PCR products were initially verified by sequencing.

**Table 3:** Table of primer sequences.

Gene Name	Gene Bank Number	Primer Sequence (5′ – 3′)	Product Size(bp)	Annealing Temperature (°C)
***Bmp4***	NM_007554	Forward AGGAGGAGGAGGAAGAGCAGReverse GAGGAAACGAAAAGCAGAGC	149	61
***Ret***	NM_001080780	Forward TAAAGCAGGCTACGGCATCTReverse GTGGTGGCAGACACAGAAGA	172	61
***Eya1***	NM_010164	Forward ACAAGTCAGGCGGTCACAGReverse CCAGGTCCCAGATGAACACT	193	61
***Gdnf***	NM_010275	Forward AATGTCCAACTGGGGGTCTAReverse GCCGAGGGAGTGGTCTTC	180	55
***Pax2***	NM_011037	Forward CTGCCCACATTAGAGGAGGTReverse TGGAAGACATCGGGATAGGA	199	55
***Six1***	NM_009189	Forward AACCCCTACCCCTCACCGReverse AGAGAGTTGATTCTGCTTGTT	177	55
***Tgfb1***	NM_011577.1	Forward AGCCCGAAGCGGACTACTATReverse TTCCACATGTTGCTCCACAC	215	55
***Tgfb2***	NM_009367	Forward TTTAAGAGGGATCTTGGATGGAReverse AGAATGGTCAGTGGTTCCAGAT	197	55
***Tgfb3***	NM_009368	Forward CGCACAGAGCAGAGAATTGAReverse GTGACATGGACAGTGGATGC	204	55
***Wnt4***	NM_009523	Forward CCGGGCACTCATGAATCTTReverse ACGTCTTTACCTCGCAGGAG	113	61
***Wt1***	NM_144783	Forward GAGAGCCAGCCTACCATCCReverse CAACTGTCAGTAGGGGTGTGG	212	61
***18s***	NR_003278.1	Forward GTAACCCGTTGAACCCCATTReverse CCATCCAATCGGTAGTAGCG	151	61

### Statistical analysis

Unpaired Student's *t*-tests, one-way or two-way ANOVAs, and Tukey's post-hoc analyses were conducted using GraphPad Prism™ 5 (Software Inc, 1992–2007). Values are mean ± SD. A probability of 0.05 or less was accepted as statistically significant.
